# The Evaluation of Xiaozeng Qianggu Tablets for Treating Postmenopausal Osteoporosis via up-Regulated Autophagy

**DOI:** 10.1155/2022/3960834

**Published:** 2022-09-19

**Authors:** Mingquan Wu, Huabing Lai, Qing Deng, Xi Peng, Jingshou Shen, Xu Zhou, Wei Peng, Liyang Zhu, He Tu, Xia Li

**Affiliations:** ^1^Department of Pharmacy, Sichuan Orthopedic Hospital, Chengdu, Sichuan, China; ^2^Department of Rehabilitation &Prosthetic Orthopedics Center, Sichuan Orthopedic Hospital, Chengdu, Sichuan, China; ^3^College of Life Science, China West Normal University, Nanchong, Sichuan, China; ^4^Department of Hands and Wrists, Sichuan Orthopedic Hospital, Chengdu, Sichuan, China; ^5^Department of Internal Medicine, Sichuan Orthopedic Hospital, Chengdu, Sichuan, China

## Abstract

**Objective:**

Postmenopausal osteoporosis (PMOP) is a common age-associated disease in the life course. Clinically, Xiaozeng Qianggu Tablets (XQT) have a potent therapeutic effect on the PMOP. However, the bioactive components and the mechanism of XQT underlying the PMOP treatment were unclear and it should be explored to discover the scientific connotation in traditional medical practice.

**Methods:**

The components in XQT were identified by UPLC-Q-TOF/MS. The animal model of PMOP was established by surgical ovariectomy in the female Sprague-Dawley rats. After treatment of XQT, the therapeutic effect was assessed by the determination of bone metabolism biomarkers in serum and histopathological examination. The effect of XQT on the autophagy and bone micro-situation were tested using western blot, RT-qPCR, and transmission electron microscope.

**Results:**

There were 27 compounds identified in XQT, including catalpol, monotropein, verbascoside, cryptochlorogenic acid, 5,7-dihydroxychromone 7-rutinoside, biorobin, and so on. The bone metabolism markers (alkaline phosphatase, bone alkaline phosphatase, procollagen type I intact N-terminal propeptide, cross-linked carboxy-terminal telopeptide of type I collagen, and tartrate-resistant acid phosphatase) were significantly increased in the PMOP rats and reversed by XQT administration. Moreover, the width of bone trabeculae and the ratio of the area of calcium deposition to bone trabeculae were also improved after treating the middle dose of XQT. Meanwhile, the bone micro-structure was improved by XQT. The mRNA and protein expression of unc-51 like kinase 1, beclin-1, and microtubule-associated protein 1B-light chain 3 in PMOP rats were down-regulated and up-regulated by XQT administration.

**Conclusions:**

The compounds in XQT, including catalpol, monotropein, verbascoside cryptochlorogenic acid, and so on, were valuable for further pharmacy evaluation. The pathological changes and bone micro-structure were improved by XQT, and the down-regulated autophagy level was also restored, which suggested a potent effect of XQT on treating PMOP, corresponding to its clinic use.

## 1. Introduction

Osteoporosis is a systemic bone metabolic disease characterized by lowered bone mass in unit volume and decreased bone strength, which increases the risk of fracture. And the main clinical manifestations are decreasing bone formation, increasing bone resorption, lost bone mass and quality, and deteriorated bone microarchitecture [1]. As the social demographic structure change due to the aging population, the incidence of primary osteoporosis is increasing year by year, being a serious health problem and burden. It has been revealed in an early meta-analysis and systematic review of 69 epidemiological investigations that the prevalence of osteoporosis was obviously increased over the past 12 years (prevalence of 14.94% before 2008 and 27.96% during the period spanning 2012–2015). The pooled prevalence of osteoporosis in people aged 50 years and older was more than twice that identified in 2006 (34.65% vs. 15.7%) and exceeded one-third of people aged 50 years and older were affected. It is worth mentioning that the prevalence of osteoporosis was higher in females than in males (25.41% vs.15.33%) and increased with age [2]. The China Health Commission released the first large-scale and multi-center epidemiological survey of osteoporosis among Chinese residents based on community population in 2018. It was suggested that osteoporosis has already become a serious health problem, especially in a middle-aged and elderly women. The incidence in people aged 50 years and older is 19.2% (female, 32.1% vs. male, 6.0%; urban area, 16.2% vs. rural area, 20.7%) and 32.0% in those aged 65 years and older (female, 51.6% vs. male, 10.7%; urban area, 25.6% vs. rural area, 35.3%). The incidence of osteoporosis in males among the Chinese population is nearly the same as in other countries. In contrast, the female incidence of osteoporosis in China is obviously higher than European and American countries [3]^.^ In addition, the number of osteoporotic fractures will reach to 6.3 million in 2050, compared with 1.7 million in 1990 [4]. And there are approximately 50% of women will experience an osteoporosis-related fracture during their lifetimes and this non-traumatic fracture underscores the need for early patient identification and continued management [5, 6]. It is particularly worth mentioning that the second phase of osteoporosis in postmenopausal women is resemble and transformed into senile osteoporosis that lasts 10–20 years with the slow bone loss [7, 8]. Stable epidemiological evidence suggested that women were more vulnerable to suffer from osteoporosis, especially PMOP, which more social care and medical concern are required.

In PMOP, the main cause of bone loss is estrogen deficiency, which directly promotes osteoclast differentiation, induces bone cell apoptosis, and inhibits the osteoblast activity. Besides, estrogen deficiency increases the expression of receptor or activator of NF-*κ*B ligand (RANKL) and macrophage colony-stimulating factor (M-SCF) secreted from bone cells and osteoblasts, reduces the level of osteoprotegerin (OPG), and further indirectly intensifies osteoclast differentiation [9]. In addition, the postmenopausal and senile females have excessive generation and accumulation of reactive oxygen species (ROS) in a higher oxidative stress status resulting from estrogen deficiency and aging, which leads to the destruction of bone micro-structure and osteoporosis [7]. The various cellular components in osteoblasts are damaged by oxidative stress and it is recognized as a crucial initiating factor for impairing osteoblastic bone formation in PMOP [10, 11]. Interestingly, the autophagy of osteocytes is inversely correlated with oxidative stress status and bone loss in the estrogen-deficient rat model induced by ovariectomy, and the therapeutic intervention of autophagy and oxidative stress could be an amenable method in clinic [12, 13]. In addition, the autophagy inhibitor chloroquine by systemic delivery can effectively reduce the osteoclastic activity and mitigate bone loss of ovariectomized mice [14]. Moreover, selective deletion of autophagy-related gene 5 (*ATG5*) in osteoclasts in *ATG5*^flox/flox^-LyzM-Cre+ mice reduce about 50% bone loss in ovariectomy-induced osteoporosis and the Atg5, Atg7, Atg4B, and LC3 proteins in autophagy were important for generating the osteoclast ruffled border, the secretory function of osteoclasts and bone resorption bone resorption *in vitro* and *in vivo.* Furthermore, Rab7, which is required for osteoclast function, localizes to the ruffled border in an Atg5-dependent manner. Thus, autophagy proteins are participated in polarized secretion of lysosomal contents into the extracellular space by directing lysosomes to fuse with the plasma membrane [15]. Similarly, glucocorticoid and ovariectomy-induced bone loss are strongly ameliorated with conditional inactivation of autophagy-related gene 7 (*ATG7*) using monocyte-specific deletion of *ATG7* in *ATG7*^fl/fl^_x_LysM-Cre mice through up-regulated osteoclast activation and differentiation [16]. Interestingly, defective autophagy in osteoblasts induces endoplasmic reticulum stress and causes remarkable bone loss [17]. Furthermore, the osteocytes apoptosis can be increased by inhibiting autophagy with estrogen deficiency, and estrogen treatment partly increases osteocyte viability by inhibiting apoptosis and maintaining autophagy [18]. Accumulating evidence support that the autophagy is important in the pathophysiology of bone cell life cycle and metabolism, including the maintenance of cell functions, differentiation, and balance between bone formation and resorption, with a conclusive role in the onset of osteoporosis and potential therapeutic value [19, 20].

The overall survival rate and quality of patients with PMOP could be improved using bone resorption inhibitors (bisphosphonates, calcitonin, estrogen, selective estrogen receptor modulators, RANKL inhibitors, and so on), bone formation accelerator (parathyroid hormone analogs), and other therapeutic mechanism agents (active vitamin D and its analogs, vitamin K2, strontium salts, and so on) following strict and standardized guidelines for clinic use. Meanwhile, the incidence of grade 3 or 4 adverse reactions (massive loss of calcium ions, induction of breast cancer or cardio-cerebrovascular diseases, vasoconstriction, and venous thromboembolism) are increasing correspondingly, and the heavy economic burden to the patient which is brought from the high price and long-term use could not be ignored [21]. The Chinese materia medica has been widely used in clinical practice with the advantages and characteristics of multi-compounds, multi-targets, and multi-approaches, which attracts worldwide attention in recent decades for its precise efficacy and relatively low toxicity and cost [22]. Xiaozeng Qianggu Tablets (XQT) is a Chinese patent medicine that was approved by Sichuan provincial Food and Drug Administration (Z20190330000), with potent therapeutic effects on tonifying liver and kidney and dispelling rheumatism. It is mainly used for treating primary osteoporosis and senile degenerative hyperosteogeny, associated with the syndrome of liver and kidney deficiency. Its direct or adjuvant therapeutic effect and safety have been fully recognized in 27-year clinical practice, with convenience in use and acceptable price. In addition, the demand from patients and the feedback from doctors and nurses were superimposed, weak and positive evidences or signal which triggered the investigation interest. Therefore, we were focused on the chemical components of XQT and the mechanism underlying its efficacy in treating PMOP in this study and tried to establish stronger evidence, which would greatly promote the popularization and secondary development of XQT.

## 2. Materials and Methods

### 2.1. Animal Handing

All animal procedures were performed in accordance with the recommendations in the Guide for the Care and Use of Laboratory Animals of the National Institutes of Health. All animal experimental procedures were approved by the Animal Experiment Ethics Committee of Sichuan Orthopedics Hospital. Female Sprague-Dawley (SD) rats at 4 months of age, commercially obtained from the experimental animal center of Sichuan University (No. SCXK (Chuan) 2018–026, Chengdu, China), were kept in cages at a barrier system and provided with a certified standard rat chow and ad libitum tap water intake. Room temperature and humidity were set at 22 ± 2°C and 50–60%, respectively, with a 12 hour light-dark cycle. One week of acclimatization was allowed prior to the experiments.

The animal model of PMOP was established by surgical ovariectomy. Each rat was fasted for 12 hours and intramuscularly injected with 50 mg/kg of Zoletil 50, a new anesthetic for animals which hardly causes intestinal flatulence and visceral adhesion after ovariectomy operation. The anesthetized rat was fixed in prone position, shaved in lumbar and dorsal, and disinfected with medical alcohol and povidone-iodine solution. An incision of about 1 cm was longitudinally operated at the junction about 1 cm from the lower edge of rib in ventral dorsal and about 1.5 cm from both sides of the spine to expose the ovary. After the fallopian tube was carefully peeled from the nearby adipose tissue and ligated, the ovary was removed. The continuous suture was used for peritoneum and muscular layer and the embedding suture was used for skin layer after resetting organs and cleaning blood stains from wound. And the rats with ovariectomy were disinfected by povidone-iodine solution and intramuscularly injected with penicillin at 40 WU for 3 consecutive days, supplied with 10% glucose in quiet and dark environment with heat preservation. The normal control rats were implemented sham operation by removing the same weight of fat around ovary. It should be noticed that self-biting by rats and pustule by infection might cause wound dehiscence, which could be avoided by wearing self-made Elizabeth circle and giving aspiration abscess treatment. All animals were kept feeding for 6 weeks.

At the 7^th^ week, the rats with ovariectomy were randomly divided into 5 groups, including PMOP rats (model group), PMOP rats treated with 0.45 mg/kg/d of estradiol valerate tablet (EVT, DELPHARM Lille S. A. S, J20171038), PMOP rats treated with 0.985 g/kg/d XQT (XQT-L), PMOP rats treated with 1.90 g/kg/d XQT (XQT-M), and PMOP rats treated with 3.8 g/kg/d XQT (XQT-H). XQT at 1.90 g/kg/d was equivalent to clinical dose for human use. The normal control and PMOP rats were received 0.9% sodium chloride solution at the corresponding volume. All medications were administered by oral gavage for 8 weeks.

All animals have fasted overnight before samples were collected. The one part of tibias in hind legs were stripped out with fibula removed and stored in 4% paraformaldehyde or 2.5% glutaraldehyde solution. Another part of tibias was stored in liquid nitrogen. The blood was collected from abdominal aorta with or without accelerating coagulation. The blood was centrifuged at 3000 rpm for 10 min at 4°C to separate the serum. And the serum was stored at −80°C before testing.

### 2.2. Isolation and Identification of Chemical Constituents by UPLC/ESI-Q-TOF/MS

The powder of XQT (1.0 g) was accurately weighed and added to 25 mL methanol and the mixture was ultrasonically extracted for 30 min at 250 W and 40 kHz. Its reduced weight was made up of 50% methanol after being mixed well and statically placed. After filtration, the filtrate was further centrifuged at 13 000 rpm for 15 min and the supernatant was filtered using a 0.22 *μ*m microporous filter member and injected into UPLC/ESI-Q-TOF/MS system. The UPLC was performed in a 100 mm × 2.1 mm, 1.7 *μ*m Waters Acquity UPLC® BEH C_18_ column (Waters Corp., Milford, USA). The mobile phase was water with 0.1% formic acid (A) and acetonitrile with 0.1% formic acid (B). The gradient elution was as follows: 3% B for 0–2 min, 3%–5% B for 2–4 min, 5%–20% B for 4–20 min and 20%–28% B for 20–28 min with flow rate of 0.4 mL/min. The injection volume of 1 *μ*L at column temperature of 40°C. The Waters SYNAPY G2HDMS system was used for mass spectrometry with ion source of electrospray ionization (ESI). The negative ion scanning (ESI^−^) modes were selected because the iridoid glycosides and styrene glycosides were the active ingredients in XQT based on previous study. The nitrogen was chosen as atomized and conical gas. The source temperature and cone gas flow rate were set at 100°C and 40 L/h. Meanwhile, the desolvation temperature and gas flow rate were set at 350°C and 800 L/h. Sampling cone voltage was set as 40 V, extraction cone voltage was 4 V, and capillary voltages was 2.5 kV (ESI^−^). The scan time and inter-scan time was 0.3 s and 0.02 s, respectively. The range of scanning mass-to-charge ratio (*m/z*) was 50–1200. The leucine-enkephalin (0.5 *μ*g/mL) at 5 *μ*L/min was used for calibration of mass number (*m/z* 554.2615 for ESI^−^).

### 2.3. Blood Chemistry Analysis

The bone turnover markers in serum, including alkaline phosphatase (ALP), bone alkaline phosphatase (BALP), procollagen type I intact N-terminal propeptide (PINP), cross-linked carboxy-terminal telopeptide of type I collagen (CTX-I), and tartrate-resistant acid phosphatase (TRAP), were measured by enzyme-linked immunosorbent assay (ELISA) method using the microplate reader (Thermo Fisher, Multiskan FC). All experiments were performed according to the manufacturer's protocol.

### 2.4. Histopathological Observation and Evaluation

The fibulas stored in 4% paraformaldehyde were rinsed by pure water in 30 min before tissue trim and were dehydrated in corrected order of 75% ethanol for 6 h, 85% ethanol for 10 h, 95% ethanol for 4 h, and absolute ethanol for 2 h with the operation of repeat twice, permeabilized with dimethylbenzene twice for 20 and 15 min, respectively, and infiltrated in paraffin for 3 h. The embedded tissue was sliced to 5 *µ*m (Leica, RM 2235) and uploaded on the glass slide via flatten in 37°C water bath and further baked at last 2 h. The section was stained which was processed by dewaxed with dimethylbenzene, rinsed with pure water for 20 min, stained with hematoxylin for 30 min, rinsed with pure water for 20 min, differentiated with 1% hydrochloric acid-alcohol, stained with eosin for 5 min, dehydrated with similar gradient alcohol, permeabilized with dimethylbenzene, and packaged with resin glue. Correspondingly, the section was stained with toluidine blue for 15 min. The changes in bone trabecula, growth plate, bone marrow cavity, and calcium deposition of tibia were observed and recorded under optical microscope.

### 2.5. RNA Extraction and Reverse Transcription Quantitative PCR

The total RNA of 50–100 mg proximal tibia was extracted using RNAiso Plus kit (Takara, 9109) and reversely transcribed using the Transcriptor First Strand cDNA Synthesis Kit (Roche, 04897030001). The mRNA expression level was quantitated using real-time fluorescence quantitative polymerase chain reaction (PCR) system (BioRad, CFX96) and Stormstar SybrGreen qPCR Master Mix kit (DBI Bioscience, DBI-2143). The primers for *β*-actin, ULK1, Beclin-1 and LC3B were designed via Primier 5 software. The species examine was performed in Primer-Blast of NCBI (https://www.ncbi.nlm.nih.gov/tools/primer-blast/) and the synthesised primers were shown at Table 1. The gene expression values were calculated based on 2^−ΔΔC*t*^ methods using the mean of the respective cytokines in mock-treated rat as the calibration. The relative quantities (RQs) were determined using the following equation: RQ = 2^(−ΔΔC*t*)^.

### 2.6. Western Blot for ULK1, Beclin-1 and LC3B

The approximate 3–5 mm proximal tibia, collected after removing soft tissue and cartilage in bone end, was grounded into powder in liquid nitrogen for protein extraction. The tissue lysate buffer was pre-prepared and pre-cooled using radioimmunoprecipitation assay (RIPA, BOSTER, AR0102) and phenylmethanesulfonyl fluoride (PMSF, BOSTER, AR1178) at volume ratio of 100 : 1. The 60 mg powdered bone was added into 600 *μ*L tissue lysate buffer to homogenate and centrifuged at 12000 rpm at 4°C for 20 min. The supernatant was collected and stored at −80°C. In addition, the total protein concentration was detected using a BCA protein concentration determination kit (Beyotime, P0010s). The proteins were loaded into each lane onto 10% SDS-polyacmide gelsryla (Boster, AR0138). After electrophoresis, the proteins were transferred to the polyvinylidene fluoride(PVDF) membranes which were blocked with 5% non-fat milk and washed in Tris-buffered saline containing 0.05% Tween 20 (TBST). The membranes were incubated with primary antibody overnight at 4°C, followed by incubation with horseradish peroxidase-linked secondary antibody (rabbit IgG, Cell Signaling Technology, 7074s, 1 : 2000). The primary antibodies used were as follows: anti-ULK1 antibody (Abcam, ab167139, 1 : 1000), anti-ULK1 (phospho S556) antibody (Abcam, ab203207, 1 : 1000), anti-Beclin 1antibody (Abcam, ab62557, 1 : 1000), and GAPDH (14C10) Rabbit mAb (Cell Signaling Technology, 2118S, 1 : 1000). The membrane was washed in TBST for 5 min and measuring gray value using a ChemiDOC™ MP imaging system (BIO-RAD, ChemiDOC™).

### 2.7. Transmission Electron Microscope Observation

The tibias stored in 2.5% glutaraldehyde solution after 24 h was trimmed into about 1 mm × 1 m × 1 mm cube and were rapidly transferred into decalcification solution. Then the samples were washed with PBS (pH = 7) for 3 times (30 min per time), fixed using 1% osmium tetroxide for 2 h, washed with PBS 3 times (30 min per times), dehydrated in the order of 30% acetone for 20 min, 35% acetone for 20 min, 70% acetone for 25 min, 80% acetone for 30 min, 90% acetone for 30 min, 100% acetone for 40 min twice. The tissue blocks were immersed in epoxy resin acetone solution to fully replace at 40°C for 2 h and were embedded and sliced into thin slices at 80 nm (Leica, UC7) after being saturated in absolute epoxide resin. Finally, the slices were stained with lead citrate solution and saturated uranium oxyacetate solution in 50% ethanol for 15 min, respectively, and were uploaded to observe the ultramicroscopic structures by transmission electron microscope system (Hitachi, HT7700).

### 2.8. Statistics Analysis

All data were analyzed using SPSS (Version 20.0, SPSS, Inc., Chicago, IL, USA) and GraphPad Prism (Version 8.2.0, GraphPad Software, California, USA). The differences in data between groups were assessed by one-way analysis of variance (ANOVA). Values were presented as mean ± standard deviation (SD). *P* < 0.05 was considered as statistically significant.

## 3. Results

### 3.1. The Chemical Constituents in the XQT

**Figure 1:**
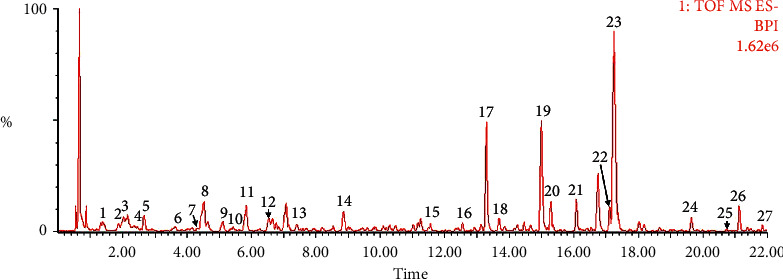
Representative negative base peak intensity (BPI) chromatograms of XQT using UPLC/ESI-Q-TOF/MS system. There were 27 compounds identified with their details displayed in Table 2.

The XQT was prepared by water extraction, alcohol precipitation and tableting process. Hence, polar compounds were the main components in the XQT, including glycosides, phenolic acids, and so on. There were 27 compounds identified from XQT in ESI^−^ mode using relative retention time, exact masses, and MS fragments, and validated by standard compounds or reported studies (Figure 1 and Table 2). The main components of XQT were iridoid glycosides, phenylpropanoid glycosides, and flavonoid glycosides, such as catalpol, monotropein, verbascoside, cryptochlorogenic acid, 5,7-Dihydroxychromone 7-rutinoside, biorobin, and so on.

### 3.2. The Observation Description of Ovariectomy-Induced Rats during Experiment Period

**Figure 2:**
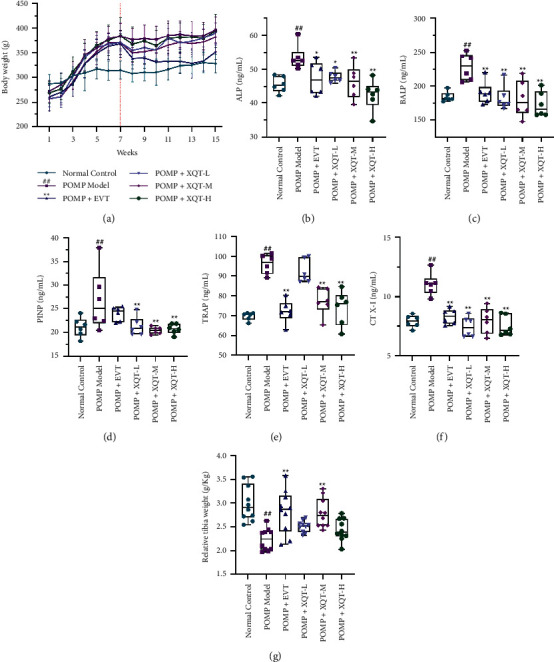
The ameliorating effects of XQT on pharmacodynamic index of PMOP. (a) The body weight monitoring of different group (*n* = 10 per group). (b)–(f) the determination of alkaline phosphatase (ALP), bone alkaline phosphatase (BALP), procollagen type I intact N-terminal propeptide (PINP), cross-linked carboxy-terminal telopeptide of type I collagen (CTX-I), and tartrate-resistant acid phosphatase (TRAP) (*n* = 6 per group). (g) The relative tibia weight (*n* = 10 per group). ^#^*P* < 0.05, ^##^*P* < 0.01 vs. normal control rats. ^*∗*^*P* < 0.05, ^*∗∗*^*P* < 0.01 vs. PMOP rats.

All rats were weighed once a week after ovariectomy or sham operation. There was no difference among all groups at the 1^st^ week (*P* > 0.05). It has been reported that animals with ovariectomy have stressfully increased body weight, while this process would not affect the progression and outcome of modeling [23]. Consistently, compared with the normal control rats, the rats who underwent ovariectomy had significant body weight gain at the 7^th^ week (*P* < 0.01). Compared with the PMOP rats, the body weight of PMOP rats treated with EVT was significantly reduced (*P* < 0.01) and was close to the level of normal control rats. And compared with the PMOP rats, only PMOP rats treated with XQT-M had obviously decreased body weight (Figure 2(a)).

### 3.3. The Amelioration of Bone Turnover Markers in Serum by XQT

Both formation and resorption of bone could be indicated by bone turnover markers, including alkaline phosphatase (ALP), bone alkaline phosphatase (BALP), procollagen type I intact N-terminal propeptide (PINP), cross-linked carboxy-terminal telopeptide of type I collagen (CTX-I), and tartrate-resistant acid phosphatase (TRAP). It was shown that the 5 bone turnover markers of PMOP rats showed a 1.17−, 1.24−, 0.27−, 1.40− and 1.38-fold increase, respectively, compared with normal control rats (*P* < 0.01), which presented the high-conversion characteristics in POMP. These bone turnover markers could be markedly down-regulated by the treatment of EVT and XQT, except TRAP in rats given with XQT-L (Figures 2(b)−2(f)). Besides, relative tibia weight was significantly increased in PMOP rats treated with EVT or XQT-M, compared with PMOP rats (Figure 2(g)).

### 3.4. The Amelioration of PMOP in the Tibia Histopathological Observation by XQT

**Figure 3:**
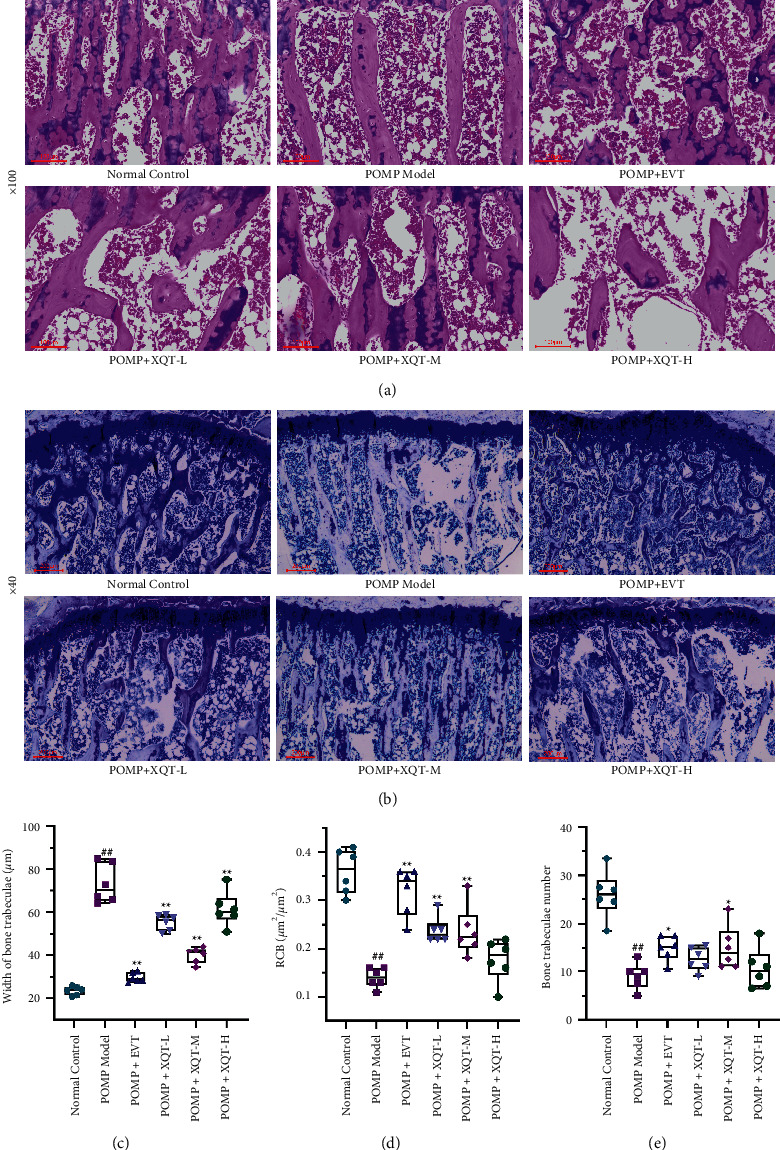
The histological improvement of PMOP by XQT. (a) and (b) were histological examination with hematoxylin-eosin or toluidine blue staining, respectively. The width of bone trabeculae (c) and ratio of the area of calcium deposition to bone trabeculae (RCB) (d) were quantitatively calculated from hematoxylin-eosin staining (*n* = 6). And the number of bone trabecular (e) was quantitatively calculated based on toluidine blue staining (*n* = 6). ^#^*P* < 0.05, ^##^*P* < 0.01 vs. normal control rats. ^*∗*^*P* < 0.05, ^*∗∗*^*P* < 0.01 vs. PMOP rats.

The protective effect of XQT on ovariectomy-induced PMOP was observed directly by histological examination with hematoxylin-eosin and toluidine blue staining. Specifically, the bone trabeculae in normal control rats were abundant, neatly arranged without fracture, and reticulated conjunction. In addition, the distances between adjacent trabeculae were small, the widths of individual bone trabeculae were uniform, and the morphology structures of bone marrow cavities were normal. The bone trabeculae in PMOP rats were lengthened and their branches were less. Meanwhile, in PMOP rats, the widths of bone trabeculae were increased, the spaces between adjacent trabeculae were enlarged which is similar to cavity, the proportion of osteoid was increased, the ratios of the area of calcium deposition to bone trabeculae (RCB) were decreased, and the bone marrow cavities were enlarged obviously. The bone loss and bone micro-structure change caused by ovariectomy could be improved by the treatment of EVT and XQT. The width of individual bone trabeculae in PMOP rats treated with EVT were slightly increased and the reticulated conjunction were disorder and irregular in contrast to normal control rats. Furthermore, the RCB were increased and the bone marrow cavities of EVT-treated rats had no obvious expansion in contrast to PMOP rats. And with the treatment of XQT, the number and branches of bone trabeculae were increased, the RCB were increased, the widths of individual bone trabeculae became narrower, and the bone marrow cavities were shrunk, compared with PMOP rats (Figures 3(a) and 3(b)). In the quantitative analysis for pathological slices, the widths of bone trabeculae could be greatly decreased by EVT or XQT, and the RCB could be apparently raised by EVT, XQT-L, and XQT-M (Figures 3(c) and 3(d)). The number of bone trabeculae in PMOP rats treated with EVT or XQT-M were also closer to normal control rats from toluidine blue staining (Figure 3(e)). Overall, the XQT-M was therapeutically potent in intervening PMOP induced by ovariectomy.

### 3.5. The Treatment of XQT Activated Autophagy

**Figure 4:**
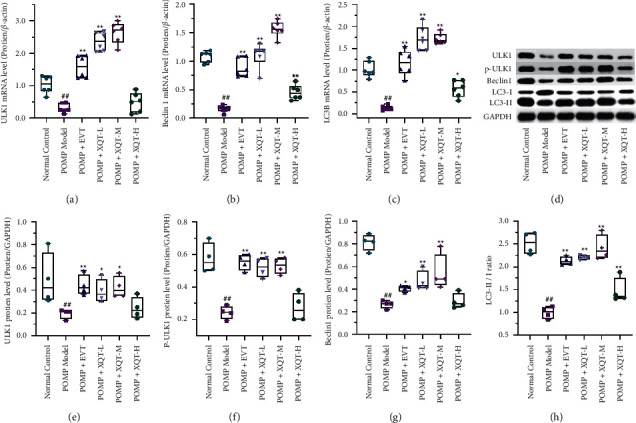
The up-regulation of the autophagy for PMOP. The mRNA levels of ULK1 (a), Beclin-1 (b), LC3B (c) and *β*-actin were detected by real-time fluorescence quantitative PCR (*n* = 6). The protein level of ULK1 (e), p-ULK1 (f), Beclin-1(g), LC3B (h) and GAPDH were analyzed by western blotting with 4 replicates (d). ULK1, Unc-51 like kinase 1. LC3B, microtubule-associated protein 1B-light chain 3. p-ULK1, phosphorylation of ULK1. ^#^*P* < 0.05, ^##^*P* < 0.01 vs. normal control rats. ^*∗*^*P* < 0.05, ^*∗∗*^*P* < 0.01 vs. PMOP rats.

Given the important role in pathophysiology of bone cell, autophagy has emerged as a potential target for the prevention and treatment of osteoporosis. Thus, the regulations of protein and mRNA expressions of the signaling pathway associated with autophagy by XQT were detected by western blot and real-time fluorescence quantitative PCR, respectively. The mRNA expressions of ULK1, Beclin-1 and LC3B in PMOP rats were significantly down-regulated, compared with normal control rats. And the treatment of EVT significantly up-regulated the mRNA levels of ULK1, Beclin-1 and LC3B, compared with PMOP rats. Moreover, the ULK1, Beclin-1 and LC3B were greatly up-regulated by the treatment of XQT-L and XQT-M (Figures 4(a)–4(c)). Correspondingly, the PMOP rats treated with EVT or XQT had notably up-regulated protein expressions of ULK1, phosphorylation of ULK1 (p-ULK1), Beclin-1, LC3B-I and LC3B-II in the proximal tibia, compared with PMOP rats, in which XQT-L and XQT-M were more effective than XQT-H (Figures 4(d)–4(h)). Therefore, XQT could upregulate the autophagy level to attenuate the PMOP induced by ovariectomy.

### 3.6. The Bone Microstructure and Autophagy Observation of Tibia from Transmission Electron Microscope

**Figure 5:**
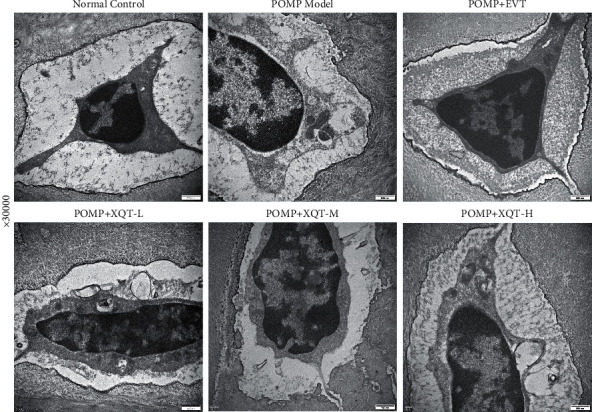
The intervention effects of XQT on POMP from transmission electron microscope in 30000 folds magnification.

The bone micro-structure and autophagosome were ameliorated by XQT treatment, which could be qualitatively reflected through transmission electron microscope. It was shown that the number of autophagosome in PMOP rats was decreased, compared with normal control rats, which could be reversed after given with EVT, XQT-L, or XQT-M, but not XQT-H. Specifically, the number of autophagosome in PMOP rats treated with XQT-L and XQT-M was likely closer to that of PMOP rats treated with EVT. In contrast, the XQT-H group was asymptotic with PMOP model rats. In respect of bone micro-structure, chondrocytes in the normal control rats were distributed singly with oval shapes. The nucleus boundary was clear, with abundant heterochromatin inside which had high electron density and relatively less euchromatin which had low electron density. The structures of organelles in the cytoplasm, such as mitochondria and endoplasmic reticulum, were clear. Three to five cell bulges were observed and their long axes were parallel to the cell. The lacunae were located between chondrocytes and bone matrix with low electron density. The cartilage lacunae around were filled with bone matrix which showed a sponge-like structure with layered and paralleled mutually, or reticulate interlaced. Compared with normal control rats, PMOP rats had slightly dilated nuclear membrane in chondrocytes. The content of euchromatin was obviously increased and the heterochromatin was decreased. It was also observed that, in PMOP rats, the cavitation areas have existed with extremely low electron density in the cytoplasm, the number of lysosomes were increased, the rough endoplasmic reticulum and mitochondria were swelled and cavitated, the boundary of the cell membrane was blurred, the cell bulges were degraded and disappeared, the cartilage matrix was disordered with irregular sponge-like loose structure, and the lacunae had no obvious morphological change with lower electron density. Compared with PMOP rats, in the PMOP rats treated with EVT, the long axis bulges were increased, the electron density in the lacuna area was increased, and the morphology of cartilage matrix was similar to normal control rats. Compared with EVT-treated rats, there were vacuolations in the cytoplasm of PMOP rats treated with XQT-L or XQT-H, but not XQT-M. Furthermore, the PMOP rats given with XQT-L or XQT-M had more abundant cell bulges, and the morphology and structure of long axis bulges were intact, compared with XQT-H or EVT-treated PMOP rats. It was notable that the arrangement of chondrocyte matrix in PMOP rats treated with XQT-L or XQT-M were regular, compared with those treated with XQT-H or EVT, while the XQT-H was relatively disorder and it was similar to the PMOP rats (Figure 5).

## 4. Discussion

Primary osteoporosis mainly includes low-conversion mode senile osteoporosis and high-conversion postmenopausal osteoporosis. There are usually no obvious clinical manifestations in low bone mass status or pre-osteoporosis phase. Moreover, there is a lack of early effective intervention or prevention due to the insufficient knowledge in public. With the deterioration of primary osteoporosis, the massive bone loss occurs, resulting into physical pain, soreness and weakness of waist with knees, deformity of spinal column, and even osteoporotic fractures. Because of the characteristics of occult onset and enormous pre- and osteoporosis population with low bone mass, PMOP was known as a soundless epidemic disease. The current prevention and treatment approaches for PMOP are mainly basic interventions (including adjusting life and basic supplementary intake for bone health), drug intervention, physiotherapy, and rehabilitation treatment. The anti-osteoporosis drugs were mainly bone resorption inhibitors (such as bisphosphonates, calcitonin, estrogens, selective estrogen receptor modulators), bone formation enhancers (parathyroid hormone) and others (active vitamin D and its analogs, vitamin K2, strontium salts). The basic therapeutic approach was the supplement of calcium and vitamin D. It was recommended that the bisphosphonates were the first choice for patients with PMOP and calcitonin was used to control osteoporosis with obvious bone pain in short term. It should be emphasized that estrogen replacement therapy (ERT) was suitable for osteoporosis patients with obvious menopausal syndrome symptoms. ERT had its unique advantages in treating PMOP, which was closely related to age, dose, administration mode, and so on. It is reported that the women who received hormone replacement therapy (HRT) including ERT before 60 years old or within 10 years after menopause would not increase the risk of breast cancer, but the application of HRT in women exceeding 60 years old or menopausal more than 10 years may increase the adverse effects to coronary heart disease [24]. In addition, estrogen should be used in early prevention or treating PMOP before 60 years old or within 10 years after menopause, especially the patients with vasomotor syndrome (VMS). A randomized controlled trial (RCT) of large samples found that lower doses of conjugated estrogen with or without medroxyprogesterone acetate could prevent the loss of bone mincral density (BMD) in spine and hip, reduce bone turnover level, and improve VMS [25, 26]. Moreover, a meta-analysis showed that estrogen transdermal patch could increase lumbar BMD after the use of 1-2 years, which could reduce the risk of venous thrombosis and atherosclerotic vascular diseases and protect the heart, compared with oral estrogen therapy [27]. Compared with estrogen therapy alone with the risk of endometrial cancer, estrogen-progestin therapy (EPT) could significantly increase vertebral BMD and may benefit cardiovascular system, which still ultimately increases the risk of breast cancer [28, 29]. And the risk of induced venous embolism, endometrial cancer, breast cancer, or stork, should also be paid attention. In the contrary, the complementary and alternative medicine, especially the traditional Chinese medicine, had less adverse reactions and may be more suitable for long-term medication to manage PMOP [30]. In this study, the ovariectomy rats had body weight gained, the relative tibia weight dropped, disorder of serum bone metabolism markers and obvious bone histopathological damage, in which the down-regulated autophagy was one of important etiology mechanism for PMOP. These manifestations could be relieved via restoring the balance between bone formation and resorption and mediating autophagy.

In the detailed analysis, XQT consists of 11 Chinese herbs, including Shudihuang, Luxiancao, Roucongrong, Jixueteng, Gusuibu, Gouji, Duhuo, Haitongpi, Shanzha, Jianqu, and Maiya which were processed with water extraction, ethanol precipitation and tableting with starch and dextrin. The complex chemical composition of XQT is the solid basis of exerting bioactivities via multi-targets and multi-approaches. The identified 27 chemical compositions almost had extensive biological activities, in which the phenylethanoid glycosides, iridoid glycosides, flavonoids or their glycosides and organic acids should be paid closed attention to in treating PMOP. Preclinical evidences showed that monotropein could attenuate ovariectomy and LPS-induced bone loss in mice, decrease inflammatory impairment on osteoblast through blocking activation of nuclear factor *κ*B (NF-*κ*B) pathway, and regulate the oxidative stress of osteoblast cells via Akt/mTOR-mediated autophagy [31, 32]. Catalpol could protect mice from inflammation- and ovariectomy-induced bone loss by inhibiting osteoclast activity. The underlying mechanism was that catalpol could upregulate phosphatase and tensin homolog (PTEN) activity by reducing its ubiquitination and degradation, and subsequently suppressing RANKL-induced NF-*κ*B and AKT signaling pathways, leading to the inhibition of NFATc1 expression [33]. In addition, the loganic acid could prevent bone mineral density loss and improve bone structural properties in ovariectomized mice [34]. Interestingly, the phenylethanoid glycosides could bind with estrogen receptor and show estrogen-like activity with two-way regulation behavior [35]. The phenylethanoid glycosides have mimetic estrogen effects at a low level of estrogen, but exert anti-estrogen activity at a high level of estrogen. Therefore, the phenylethanoid glycosides are called selective estrogen receptor modulators. There were five reported promising anti-osteoporotic candidates (including echinacoside, jionoside A1, verbascoside, isoacteoside and 2′-acetylacteoside) for intervening osteoporosis. Echinacoside stimulates bone regeneration in MC3T3-E1 cells *in vitro* and has a remarkable anti-osteoporotic activity in ovariectomized rats [36]. Jionoside A1 attenuates bone loss and improves bone quality in ovariectomized rats partly through its regulation of the canonical Wnt/*β*-catenin signaling pathway [37]. Verbascoside (Acteoside) suppresses RANKL-mediated osteoclastogenesis by inhibiting c-Fos induction and NF-*κ*B pathway and reducing ROS production [38]. The isoacteoside increases the growth and differentiation of MC3T3E1 cell [39]. Similarly, the therapeutic effect of 2′-acetylacteoside on bone resorption of ovariectomized mice is mainly related to the regulation of RANKL/RANK/TRAF6-mediated NF-*κ*B/NFATc1 pathway, which is confirmed by the down-regulated expressions of RANK, TRAF6, I*κ*B kinase *β*, NF-*κ*B and NFATc1 [40]. It is demonstrated that flavonoids and their glycosides have potential applications for osteoporosis treatment. Hyperoside could rectify bone metabolism disorder in ovariectomized mice, which was related to the inhibition of the TRAF-6 mediated RANKL/RANK/NF-*κ*B signaling pathway and the elevation of the OPG/RANKL ratio [41]. Naringin has promising prospect in the treatment of bone disorders, such as osteoporosis and osteoarthritis, for its exciting pro-osteogenic and antiresorptive properties in extensive osteoporosis models [42]. It was highlighted that quercetin could inhibit RANKL-mediated osteoclastogenesis, osteoblast apoptosis, oxidative stress, and inflammatory response via promoting osteogenesis, angiogenesis, antioxidant expression, adipocyte apoptosis and osteoclast apoptosis. The possible underlying mechanisms are regulation of Wnt, NF-*κ*B, Nrf2, SMAD-dependent, and intrinsic and extrinsic apoptotic pathways. On the other hand, quercetin exerted complex and competing actions on the MAPK signaling pathway to orchestrate bone metabolism, resulting in both stimulatory and inhibitory effects on bone in parallel. The overall interaction is believed to result in a positive effect on bone [43]. Protocatechuic acid prevents osteoclast differentiation by regulating oxidative stress and inflammation and inducing apoptosis in murine macrophage cells *in vitro*, and attenuates trabecular and inflammatory bone loss in ovariectomized mice [44–46]. Moreover, chlorogenic acid had cytoprotective effect against hydrogen peroxide-induced oxidative stress and it promoted the Nrf2/HO-1 anti-oxidative pathway by activating p21 ^Waf1/Cip1^ to prevent Dex-induced mitochondrial apoptosis in osteoblastic cells [47, 48]. Generally, the XQT exhibited good anti-postmenopausal osteoporosis activity.

In cellular environments, the induction of autophagy requires the repression of the mTOR kinase which, when active, inhibits autophagy by phosphorylating and inactivating the kinase unc-51-like kinase 1 (ULK1) and another component of the kinase complex ATG13. When mTOR is inhibited, changes in the phosphorylation of ULK1, ATG13, and other proteins in the complex, including RB1-inducible coiled-coil 1 (RB1CC1, also known as FIP200), stimulate ULK1 activity and induce autophagy. In addition, the mTOR is negatively regulated by AMPK, of which expression is up-regulated with suppressed autophagy. The signaling pathways of PI3K-Akt, MAPK-Erk1/2 or p53/genotoxic stress also regulated mTOR activity to induce or inhibit autophagy. In this study, we discovered that the mRNA and protein expressions of ULK1, Beclin-1, and LC3B in PMOP rats were down-regulated and could be reversed after treatment of XQT. Combined with the improvement of morphological and serum bone metabolism index, the PMOP could be intervened by XQT, which might be related to reducing oxidative stress and inflammatory damage of osteocytes and restoring the dynamic equilibrium between bone formation and bone resorption via up-regulated autophagy. Comprehensively, the XQT is a valuable Chinese patent medicine to treat PMOP for in-depth pharmaceutical development and mechanism elucidation.

## 5. Conclusions

The middle dose group was equivalent to the clinic using dose of XQT which had the best performance in treating PMOP, and the bone micro-structure was improved, and the autophagy level also be up-regulated significantly. It was comprehensively suggested that treating PMOP given XQT-L or XQT-M may be more beneficial to POMP patients. XQT should be regarded as a valuable anti-postmenopausal osteoporosis candidate for application, investigation, and further development.

## Figures and Tables

**Table 1:** Forward and reverse primers used in reverse transcription quantitative PCR.

Gene ID	Target gene	Primer sequence (5′-3′)	Tm (°C)	Product length (Bp)
NM_031144.3	*β*-actin:F	ACAACCTTCTTGCAGCTCCTC	60	200
	*β*-actin:R	CTGACCCATACCCACCATCAC		

NM_001108341.1	ULK1:F	ATGGTGGAGACCTGGCTGAC	52.5	203
	ULK1:R	AATCTTGACTCGGATGTTGCTG		

XM_017596950.1	Beclin-1:F	TGAGGAGCAGTGGACAAAGG	60	75
	Beclin-1:R	TGTGAGGACACCCAAGCAAG		

NM_022867.2	LC3B:F	AGAGCGATACAAGGGTGAGAAG	57.1	134
	LC3B:R	AGAAGGCTTGGTTAGCATTGAG		

The all primers were rat species. F, Forward primer, R, Reverse primer. Tm, Temperature of melting.

**Table 2:** ESI-TOF/MS data for compounds identified in Xiaozeng Qianggu tablets.

No.	Retention time (min)	Addition mode	Molecular formula	Molecular weight	Error in ppm	Compound name	Structures	Composition attribution
1	1.34	[M-H]^−^	C_6_H_6_O_3_	125.0240	0.8	5-Hydroxymethylfurfural	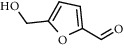	Jianqu and Maiya
2	1.86	[M-H]^−^	C_15_H_22_O_10_	361.1140	1.4	Catalpol	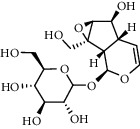	Shudihuang
3	1.88	[M-H]^−^	C_16_H_22_O_11_	389.1078	−1.5	Monotropein	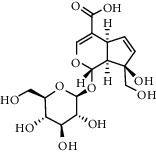	Luxiancao
4	2.43	[M-H]^−^	C_27_H_42_O_20_	685.2184	−1	Rehmannioside D	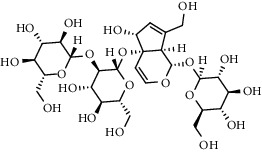	Shudihuang
5	2.67	[M-H]^−^	C_7_H6O_4_	153.0190	1.3	Protocatechuic acid	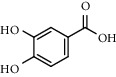	Gouji and Haitongpi and Jixueten
6	3.63	[M-H]^−^	C_16_H_22_O_10_	373.1140	1.3	Geniposidic acid	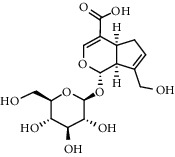	Roucongrong
7	4.34	[M-H]^−^	C_7_H_6_O_3_	137.024	0.7	Protocatechualdehyde	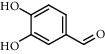	Gouji
8	4.53	[M-H]^−^	C_15_H_18_O_9_	341.0881	2.3	1-O-caffeoyl-*β*-D-glucopyranose	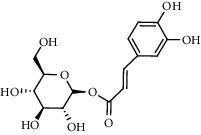	Gusuibu
9	5.12	[M-H]^−^	C_16_H_24_O_10_	375.1289	−0.5	8-Epiloganic acid	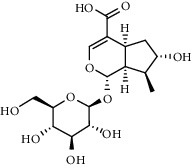	Roucongrong
10	5.42	[M-H]^−^	C_20_H_30_O_12_	461.1660	0.2	Decaffeoylacteoside	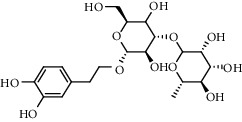	Roucongrong
11	5.84	[M-H]^−^	C_16_H_24_O_10_	375.1289	−0.5	Loganic acid	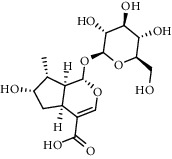	Roucongrong
12	6.55	[M-H]^−^	C_16_H_18_O_9_	353.0880	2	Chlorogenic acid	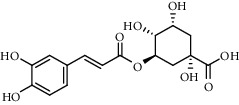	Roucongrong and Shanzha and Haitongpi
13	7.4	[M-H]^−^	C_16_H_18_O_9_	353.0880	2	Cryptochlorogenic acid	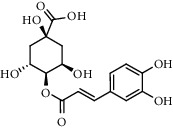	Roucongrong & Shanzha and Haitongpi
14	8.87	[M-H]^−^	C_21_H_26_O_13_	485.1301	1.2	5,7-Dihydroxychromone 7-rutinoside	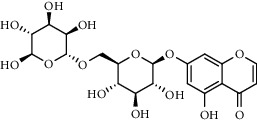	Gusuibu
15	11.56	[M-H]^−^	C_27_H_30_O_15_	593.1506	0.5	Biorobin	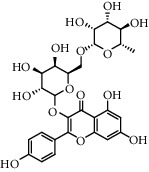	Gusuibu
16	12.56	[M-H]^−^	C_35_H_46_O_20_	785.2514	−0.1	Purpureaside C	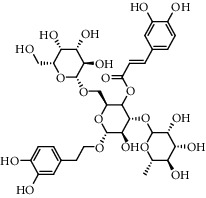	Shudihuang
17	13.29	[M-H]^−^	C_35_H_46_O_20_	785.2514	−0.1	Echinacoside	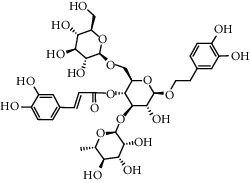	Shudihuang & Roucongrong
18	13.67	[M-H]^−^	C_21_H_20_O_12_	463.0876	−0.2	Hyperoside	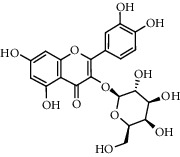	Luxiancao and Shanzha
19	15	[M-H]^−^	C_27_H_32_O_15_	595.1669	1	Neoeriocitrin	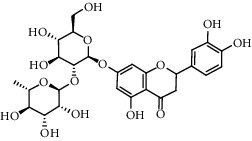	Gusuibu
20	15.29	[M-H]^−^	C_36_H_48_O_20_	799.2651	−1.3	Jionoside A1	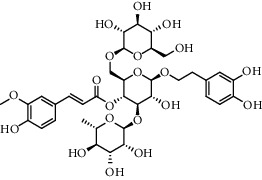	Shudihuang
21	16.09	[M-H]^−^	C_29_H_36_O_15_	623.1961	−2.4	Verbascoside	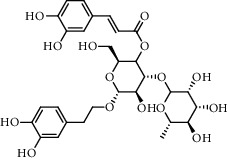	Shudihuang and Roucongrong
22	17.12	[M-H]^−^	C_29_H_36_O_15_	623.1961	−2.4	Isoacteoside	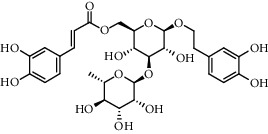	Shudihuang & Roucongrong
23	17.26	[M-H]^−^	C_27_H_32_O_14_	579.1709	−0.9	Naringin	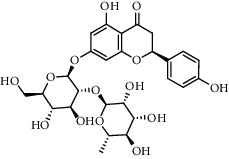	Gusuibu and Shanzha
24	19.65	[M-H]^−^	C_31_H_38_O_16_	665.2095	2	2′-acetylacteoside -	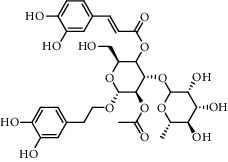	Roucongrong
25	20.94	[M-H]^−^	C_15_H_10_O_7_	301.0351	1	Quercetin	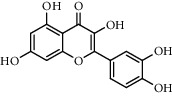	Luxiancao and Shanzha
26	21.12	[M-H]^−^	C_31_H_38_O_16_	665.2095	2	Tubuloside B	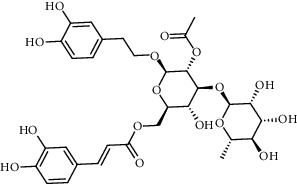	Roucongrong
27	21.85	[M-H]^−^	C_31_H_40_O_15_	651.2300	1.7	Martynoside	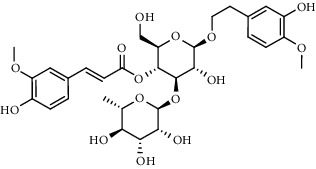	Shudihuang

## Data Availability

The datasets used and analyzed during the current study can be obtained from the corresponding author on reasonable.
